# Base Modifications: Regulation of Stem Cell Functions and Diseases

**DOI:** 10.1155/2018/5402384

**Published:** 2018-12-09

**Authors:** Yujing Li, Changwon Park, Xuekun Li

**Affiliations:** ^1^Emory University, Atlanta, USA; ^2^Zhejiang University, Zhejiang, China

Vast emerging lines of evidences support importance of the crosstalk between base modifications and stem cell functions such as lineage commitment, specification, self-renewal, quiescence, proliferation, and differentiation. The main forms of base modifications in DNA and RNA (coding and noncoding RNA) are N6-methyladenosine (m6A) and 5-methylcytosine (5mC) and its oxidative derivatives 5-hydroxymethylcytosine (5-hmC), 5-formylcytosine (5-fC), and 5-carboxylcytosine (5-caC). The delicate spatiotemporal alteration of base modifications constitutes an integral part of controlling mechanisms of self-renewal, ensure quiescence, proliferation, and differentiation, which is critical for ensuring successful development and maintaining postnatal life.

Over the past decades, significant progress has been made in appreciation of the link between aberrant base modifications in stem cells and diseases, such as cancers and neurodegenerative disorders. These achievements keep inspiring scientists to further uncover the epigenetic mechanisms for stem cell development and to dissect pathogenesis of the multiple diseases conferred by developmental dysfunctions of the stem cells. The special issue “Base Modifications: Regulation of Stem Cell Functions and Diseases” mainly focuses on the original research and review articles that address the recent advances in base modification-mediated regulation of stem cell functions and their link with the diseases.

In this special issue, H. Tao et al. investigated the expression pattern of ten-eleven translocation proteins (TETs) which catalyze the oxidation of 5-mC to 5-hmC and further to 5-fC and 5-caC and the levels as well as the distribution of the 5-hmC, 5-fC, and 5-caC in postnatal neurons and adult neuronal stem cells (aNSCs). The authors demonstrated that the three distinct forms of the modified bases and their writer TETs are highly enriched in multiple brain regions and in the aNSCs and display temporal and spatial patterns during the postnatal neuron development, contributing to dynamic epigenetic regulation of the related gene expression.

In a separate review article, H. Zhou et al. highlighted the recent advances in the critical roles of TETs, the 5-mC and 5-hmC markers, noncoding RNAs, and histone modifications in proliferation and differentiation of the NSCs and tumorigenesis. Furthermore, the article addressed the potential link between the aberrant 5-hmC modification and the neurological disorders such as Alzheimer's disease (AD), Huntington's disease (HD), Rett syndrome, and major depressive disorders (MDD). Finally, the perspective is given regarding the future approaches to comprehensively understand the epigenetic roles that the 5-hmC marker and its writer TETs play in the neurological disorders as well as to discover the potential therapeutic targets for these disorders.

N6-Methyladenosine in both DNA and RNA has also attracted significant attention due to its essential regulation roles in the expression of genes involved in stem cell fate, CNS development and functions, and some diseases including cancers. A. Shah et al. reported that DDX3, a member of the family of DEAD box RNA helicases, functions as a regulator for ALKBH5 which can catalyze demethylation of the m6A in mRNAs. A series of experiments revealed that the regulation of DDX3's function is dependent upon the interaction between the ATP domain of DDX3 and the DSBH domain of ALKBH5 which has been acknowledged as a main m6A RNA demethylase. In addition to the regulation of the mRNA demethylation, DDX3 could regulate the methylation status of microRNAs through the interaction with AGO2 as well. This discovery adds one more clue to understand the regulation of ALKBH5 by other components as well as the crosstalk between N6-methyladenosine modification and the microRNA pathway.

A review article by P. Ji et al. systematically highlighted the research progress in N6-methyladenosine as an epitranscriptomic and epigenetic player implicated in determination of stem cell fate. Firstly, the article summarized the characteristics of the N6-methyladenosine marker complexes including the writers, the erasers, the readers, and other regulation components at the biochemical, molecular, and phenotypical levels. Then, the potential clinical applications of the components in the complexes as the therapeutic targets were discussed. Furthermore, the article summarized the dynamic regulation and fine-tuned coordination of the levels and the landscapes of the N6-methyladenosine marker in accordance with stages of the growth, development, and reproduction as naturally programmed during the lifespan. More specifically, the article addressed the association between aberrant m6A modification in stem cells and initiation/development of the diseases such as cancers. Finally, the future research directions are discussed.

Expanded further from the base modification, N. Xie et al. emphasized the advances in the CRISPR/Cas9-based novel epigenetic modulation techniques. Starting from the epigenome editing, the first part of the article discussed the rewriting of the epigenetic markers at the given loci in the genome to uncover the effects caused by the targeted rewriting. This epigenome editing strategy could also confer the potential application to screening and annotation of the epigenetic elements such as enhancers. Furthermore, the article summarized the research progress in temporal and spatial control of epieffectors induced by light and chemicals in combination with the CRISPR/Cas9 system to determine cell phenotypes during development, aging, and disease pathogenesis. Besides, the interesting and the valuable progress in the CRISPR/Cas9-based characterization of the chromatin structures and the interactions were addressed. Finally, the article addresses the current drawbacks and the future directions including the potential clinical applicability.

Other than studies on base modification study, M. Mellado-López et al. reported two important functions of Plasma Rich in Growth Factors (PRGF) in adipose-derived stem cells (ASCs). Firstly, the PRGF could favor survival and proliferation of human and canine ASCs and delay human ASC senescence and autophagocytosis in comparison with serum-containing cultures. Additionally, PRGF could efficiently enhance the differentiation of the canine- and human-derived ASC into osteocytes, adipocytes, or chondrocytes. The PRGF-induced AKT phosphorylation to avoid the lethal concentrations of hydrogen peroxide was proposed as the mechanism for prevention of ASC death. It is well known that ASCs have become a promising therapeutic alternative for tissue repair in various clinical applications. Given that the conventional serum-based ASC culture methods confer the restrictive cell survival, differential tissue integration, and undirected cell differentiation after transplantation in a hostile microenvironment, this discovery could be a breakthrough for ASC culture and future SC-based regenerative medicine.

The authors of this special issue hold strong wishes that the research and the review articles published here, technically and scientifically, could provide important information more conveniently for the readers with strong interest in the closely related research field.

